# A method to predict overall food preferences

**DOI:** 10.1371/journal.pone.0268520

**Published:** 2022-06-03

**Authors:** Vilis O. Nams, Matt W. Hayward

**Affiliations:** 1 Department of Plant, Food and Environmental Sciences, Faculty of Agriculture, Dalhousie University, Truro, Nova Scotia, Canada; 2 School of Environmental and Life Sciences, University of Newcastle, Callaghan, New South Wales, Australia; 3 Mammal Research Institute, University of Pretoria, Hatfield, Pretoria, South Africa; University of Fribourg, SWITZERLAND

## Abstract

Most natural ecosystems contain animals feeding on many different types of food, but it is difficult to predict what will be eaten when food availabilities change. We present a method that estimates food preference over many study sites, even when number of food types vary widely from site to site. Sampling variation is estimated using bootstrapping. We test the precision and accuracy of this method using computer simulations that show the effects of overall number of food types, number of sites, and proportion of missing prey items per site. Accuracy is greater with fewer missing prey types, more prey types and more sites, and is affected by the number of sites more than the number of prey types. We present a case study using lion (*Panthera leo*) feeding data and show that preference vs prey size follows a bell-curve. Using just two estimated parameters, this curve can be used as a general way to describe predator feeding patterns. Our method can be used to: test hypotheses about what factors affect prey selection, predict preferences in new sites, and estimate overall prey consumed in new sites.

## Introduction

Most natural ecosystems contain animals feeding on many different types of food, but it is difficult to predict what will be eaten when food availabilities change. Ecologists face this issue all the time. For example, if we introduce a biocontrol agent then will it escape regulation by predators? Or if climate change shifts the range of a plant species then how will that affect feeding on other plants eaten by a common herbivore? Or if an invasive species appears then how will its predators respond? We need to be able to predict food preference for any combination of food types.

At the moment, theories of predator behaviour explain processes in current study sites but do not predict preference in new study sites. Such theories include optimal foraging [1–3] and more specific theories on frequency-dependent predation [4, 5], prey-switching [6, 7], ideal-free distributions [8, 9], and functional responses [10–12]. These theories were not developed to predict preferences in new study sites.

There have also been some excellent studies looking at how dietary preference varies with factors such as amounts of food [7, 13], seasonality [14, 15] or habitat [16, 17]. However, these studies have all been limited to narrow conditions. It has been difficult to predict on a large scale how preference varies with density or habitat or season, because it is difficult to disentangle effects of those factors vs the effects of other food types. One of us (MWH) has simply averaged preferences over many sites, assuming that large sample sizes from diverse food communities across large geographic areas will account for the challenges of comparing preferences between diverse communities [18–20].

One main reason for this general paucity in predicting preferences is methodology. While there is a mature field of study about ways to estimate prey preference in one study site [21–25], there is no method to combine preference estimates from many study sites and then predict preference in a new study site that has a unique combination of prey types.

The difficulty in combining prey preferences over many sites arises from the fact that all measures of preference are relative to the prey items present at individual sites. Just adding or subtracting one prey type may completely change preferences for the other prey; thus, it is difficult to combine preferences among sites that have different types of prey.

For example, suppose a predator prefers 4 prey items in this order A>B>C>D. In a site containing only prey items C & D, the predator would select for C. In a site containing prey items A, B, C, the predator would select against C. It is not known how to combine data from those 2 sites to predict selection when all prey are present. One would estimate that both C and B have intermediate selection values but it is not known how to estimate overall preference for B vs C. The problem is much more difficult when 20 prey species exist in 40 different sites.

Previous studies have calculated prey preference values for each prey type within each study site, then averaged these individual preference values over all study sites for each prey type [26, 27]. The problem with this is that prey preference estimates within one study site are relative to the prey types present and thus cannot be directly combined with other study sites. Johnson [28] developed a method to combine preferences estimates, but rather than estimating a specific preference, the method estimated a rank for each prey item.

We present a method that estimates overall prey preference by combining data over many study sites, even when not all sites contain all prey items. Our method adjusts for the relative nature of preferences at individual study sites, allowing one to predict feeding preferences in new study sites, or when prey items change. This method also produces estimates of sampling variation, and thus, confidence intervals for preference estimates. We show how to increase precision by weighting the estimates by both prey densities and sampling effort. We then illustrate the method with a simple numerical example and test the method’s accuracy and precision using simulations. Finally, we show an example application using data from lion (*Panthera leo*) feeding.

## The proposed method

**Fig 1:**
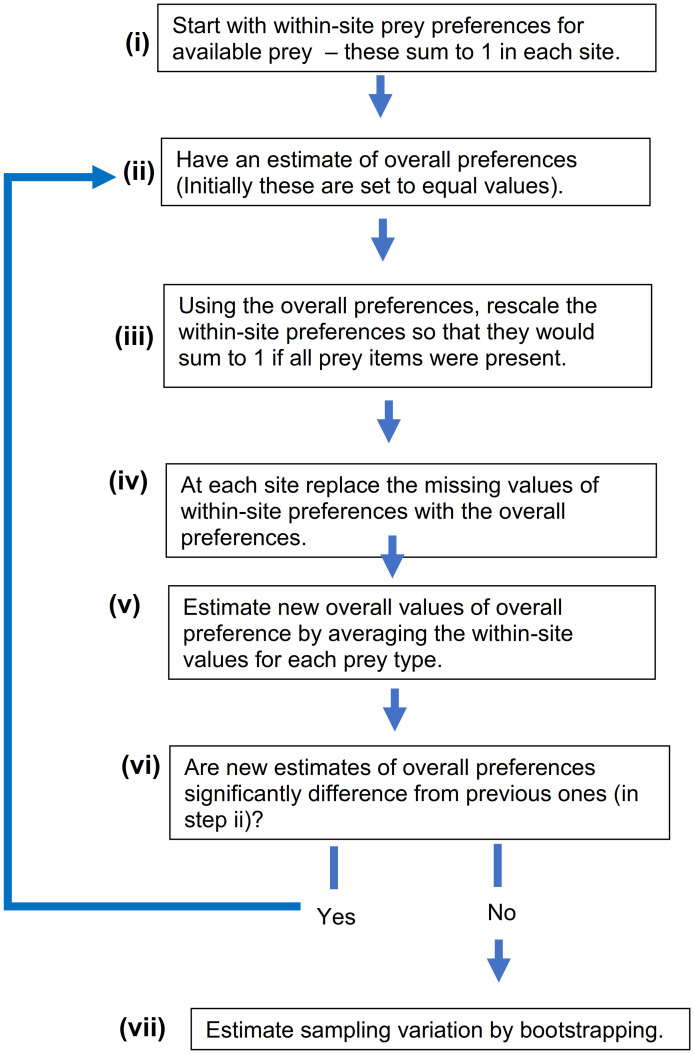
Overview of method.

The aim is to estimate overall feeding preference by one species of animal, based on data from more than one study site. Since our method is based on iteration, we will call this the Iterative Preference Averaging (IPA) method. Note that, following convention, we refer to foods as “prey”, and the feeder as the “predator”. However, our method applies to all types of animals, not just predators, and the selected items could be vegetation, or even non-food items, such as types of habitat. All analyses were carried out using the Wolfram Language [29], and R code for the final method is given in Nams & Garnett [30]. See Fig 1 for an overview.

### Data input

At each site there is a list of prey items that the predator eats, and for each prey item we have estimates of abundance and consumption. Abundance estimates can originate by any means (e.g. transect counts [31], capture-recapture [32], or track counting [33]), and do not have to be absolute (i.e. one can use indices of abundance). Similarly, the measures of prey consumption can originate by any means (e.g. DNA analysis of stomach contents [34], direct counts of the number of kills [35], or hair identification from scat contents [36]). Prey abundance and consumption estimates can be obtained by different methods at each site, as long as the same methods are used at each site. Thus, IPA is a robust method that can be used for meta-analyses combining results of many studies.

### Details

Let m = number of sites,

n = overall number of prey items,

d_ij_ = density of prey item j at site i

e_ij_ = amounts of prey item j consumed at site i.

**Step (i)**. At each site we estimate within-site preferences for those prey that are available:

$$\propto_{\text{ij}}=\frac{\frac{e_{\text{ij}}}{d_{\text{ij}}}}{\sum_{j}\frac{e_{\text{ij}}}{d_{\text{ij}}}}$$ (1)

Note that the ∝_ij_ all sum to 1 for each site, and j is summed over all prey types present in site i, (not all prey are available at each site). This preference estimate is the Manly’s ∝ [22, 23].

We want to estimate:

*p*_j_ = overall prey preference for prey item j. This is equivalent to an overall ∝_j_ when all prey types are present.

We explain the procedure with an example.

Suppose we have m = 4 sites and n = 6 prey types. The within-site preferences (∝) are given by the following matrix, where columns are prey types and rows are sites. The dashes show when the prey type is not present at that site.



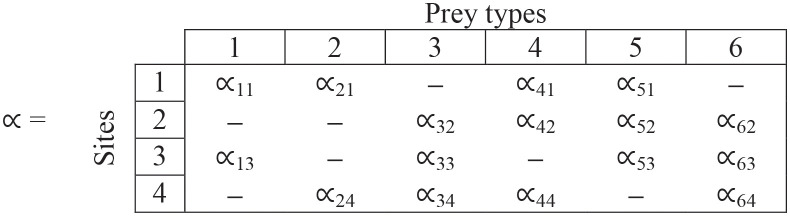

(2)


**Step (ii)**. We are estimating overall prey preferences iteratively. We start with some initial values of overall prey preference (p_i_ values), and then update these at each iteration k. For the first iteration we choose equal values that sum to 1, and for the k^th^ iteration, we use the averages summed over all sites.

**Step (iii)**. The α values at each site initially sum to 1. These are then scaled to the values you would expect if all prey were at that site—this step is the key to the whole method. This scaling is done because preferences cannot be simply averaged over sites, because at each site they are relative to each other—i.e. they sum to 1, even though some prey are missing. Thus, in order to combine them, we rescale the preferences so that they would sum to 1 if all prey items were present.

We calculate this scaling constant by the following. Since all prey items are not present, we replace the missing prey items with the overall prey preference estimates. Then we choose the constant so that the new preferences sum to one.

Let ${\hat{\text{p}}}_{•\text{jk}}$ = the estimate of prey preference for prey type j, averaged over all sites (i.e. overall preference), for the k^th^ iteration of the estimation process,

${\hat{\text{p}}}_{\text{ijk}}$ = the estimate of prey preference for prey type j, at site i (i.e. within-site preference), for the k^th^ iteration of the estimation process,

${\hat{P}}_{\text{k}}$ = the matrix of all preference estimates for each prey type at each site, for the k^th^ iteration of the estimation process,

${\hat{p}}_{\text{k}}$ = the vector of overall estimates of prey preference, for the k^th^ iteration of the estimation process,

$\hat{p}$ = the final vector of overall estimates of prey preference,

A_i_ = the set of prey items that are available in site i,

M_i_ = the set of prey items that are missing in site i,

c_i_ = the constant that rescales all preferences

These new preferences in each site all sum to 1—i.e.:

$$\underset{\text{j}\in {\text{A}}_{\text{i}}}{\sum }{\text{c}}_{\text{i}}\propto_{\text{ij}}+\underset{\text{j}\in {\text{M}}_{\text{i}}}{\sum }{\hat{\text{p}}}_{•\text{jk}}=1$$ (3)

If we re-arrange Eq (3), noting that the ∝_ij_ sum to one, and solve for c_i_, we get that the scaling constant for each site is

$${\text{c}}_{\text{i}}=1-\sum_{\text{j}\in {\text{M}}_{i}}{\hat{\text{p}}}_{•\text{jk}}$$ (4)

For example, in site 1 prey types {1,2,4,5} are present, and thus all of the preference values in site 1 are scaled by:

$${\text{c}}_{1}=1-\left({\hat{\text{p}}}_{•1\text{k}}+{\hat{\text{p}}}_{•2\text{k}}+{\hat{\text{p}}}_{•4\text{k}}+{\hat{\text{p}}}_{•5\text{k}}\right)$$ (5)

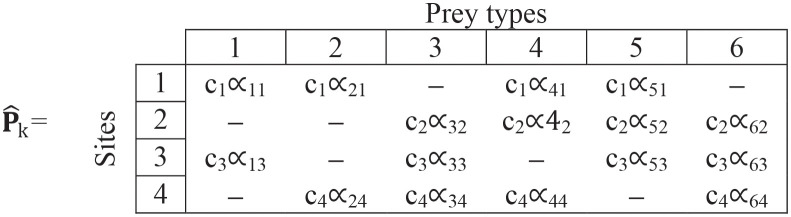
(6)

**Step (iv)**. The missing values in matrix (6) are replaced by the current estimates p_jk_, giving:

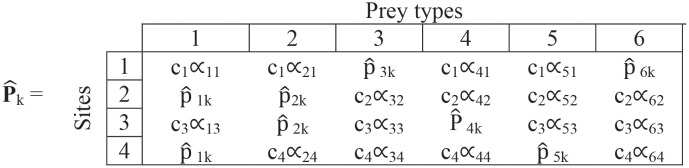
(7)

This matrix gives ${\hat{\text{p}}}_{\text{ijk}},$ the estimates for the k^th^ iteration for within-site preferences for each prey type at each site. Note that each row now sums to 1.

**Step (v)**. The overall prey preferences for the next iteration are estimated by the means of the columns in matrix (7). E.g. the overall preference for prey item 1, for the next iteration would be:

$${\hat{\text{p}}}_{1•\text{k}+1}=\frac{1}{\text{m}}\sum_{\text{j}=1}^{\text{m}}{\hat{\text{p}}}_{1\text{jk}}=\frac{1}{4}\left({\text{c}}_{1}\alpha_{11}+{\hat{\text{p}}}_{1k}+{\text{c}}_{3}\alpha_{31}+{\hat{\text{p}}}_{1k}\right)$$ (8)

**Step (vi)**. Compare the new estimate for overall preference to the one used in step (ii). If there is a change in the estimates, then repeat steps (ii)–(v) with the same initial table of preferences but with the new vector of overall prey preferences. The result is a set of overall preference estimates. These preferences range from 0 to 1 and sum to 1, and the important aspect is their values relative to each other, not their absolute values.

**Step (vii)**. Ecological estimates should always have a measure of their error [37]. This can be estimated using bootstrapping [38], treating each site as an independent sample. Briefly, this would involve taking a random number of m rows (sampled with replacement) from the matrix in Eq (2) and then estimating preferences using IPA. This would be repeated many times (typically 1,000–10,000) [38], and the mean and standard deviation values calculated from the random sample estimates. After each bootstrapping sample, the preferences should be transformed by arcsine(2 x − 1) (since prey preferences represent a proportion, and proportions are known to have variances that depend on the mean). Then means, variances, and confidence intervals would be estimated. The means and confidence intervals should be back-transformed. Note that bootstrapping is not a replacement for adequate sampling—i.e. since the sampling units are sites, using very few sites will result in low precisions (wide confidence intervals).

Preliminary simulations showed that using this transformation significantly increased accuracy of the estimates.

### Weighting

These estimates can be improved by appropriate weighting, since variances among prey preference estimates are not equal. Typically these variances are not known, however they are decreased by sampling more predators and in areas of higher prey density. For example, the preference estimate would be very variable for rare prey types. Thus we can use inverse-variance weighting [39] to minimize variation of the overall preference estimates, by weighting by prey consumption effort and/or density. Note that this can only be done when prey consumption and/or density are estimated by the same methods across all sites.

Prey consumption effort weighting is carried out as follows. For each prey type the precision of overall preference would be affected by the total number of feeding samples collected at each site. Let:

f_i_ = sample size used to estimate prey consumption—e.g. number of individuals eaten, number of scats, etc.

Weighting Eq (8) by feeding sampling effort gives:

$${\hat{\text{p}}}_{1•\text{k}+1}=\frac{\sum_{\text{i}=1}^{\text{m}}{\text{f}}_{\text{i}}\;{\hat{\text{p}}}_{\text{ijk}}}{\sum_{\text{i}=1}^{\text{m}}{\text{f}}_{\text{i}}}$$ (9)

Density weighting is carried out as follows. Let

A_i_ = the set of prey items that are available in site i, and
n_i_ = number of prey types present at site i—thus, n_i_ = |Ai|.

Since not all prey types are at each site, there are some missing values in the densities d_ij_. We handle them by imputation—i.e. by substituting the mean density of all other prey at that site. Thus, if prey type s is not present at site i, then for it’s density we use:

$${\text{d}}_{\text{is}}=\frac{1}{{\text{n}}_{\text{ij}}}\sum_{\text{j}\in {\text{A}}_{\text{i}}}{\text{d}}_{\text{ij}}$$ (10)

Weighting Eq (8) by relative prey densities gives an estimate for overall preference for prey type 1 for the next iteration of:

$${\hat{\text{p}}}_{1•\text{k}+1}=\frac{\sum_{\text{i}=1}^{\text{m}}{\text{r}}_{\text{ij}}\;{\hat{\text{p}}}_{\text{ijk}}}{\sum_{\text{i}=1}^{\text{m}}{\text{r}}_{\text{ij}}}$$ (11)

Weighting by both prey consumption effort and prey densities is carried out by weighting Eq (8) as follows:

$${\hat{\text{p}}}_{1•\text{k}+1}=\frac{\sum_{\text{i}=1}^{\text{m}}{\text{f}}_{\text{i}}{\text{r}}_{\text{ij}}\;{\hat{\text{p}}}_{\text{ijk}}}{\sum_{\text{i}=1}^{\text{m}}{\text{f}}_{\text{i}}{\text{r}}_{\text{ij}}}$$ (12)

### A numerical example

In this toy example, there are 6 prey types at 4 sites.

**Step (i)**. The values in each cell are within-site preferences—note that rows sum to 1 for each site.

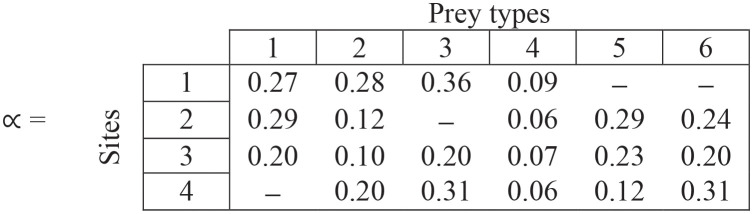
(13)

**Step (ii)**. We start with initial estimates of equal values of overall preference for the first iteration:

$${\hat{\text{p}}}_{1}=\left[0.167\;0.167\;0.167\;0.167\;0.167\;0.167\right]$$ (14)

**Step (iii)**. We rescale the ∝_ij_ values by multiplying (for each site) by Eq (4):

c=[0.667,0.833,1,0.833] (15)

to get:

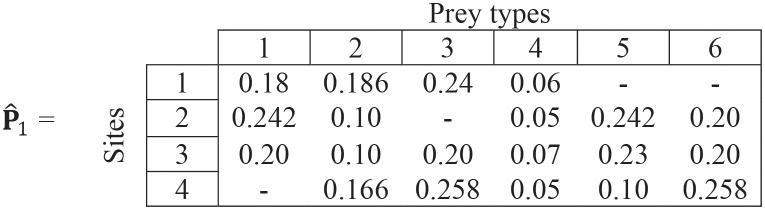
(16)

**Step (iv)**. We replace the missing values by the overall preferences from Eq (14):

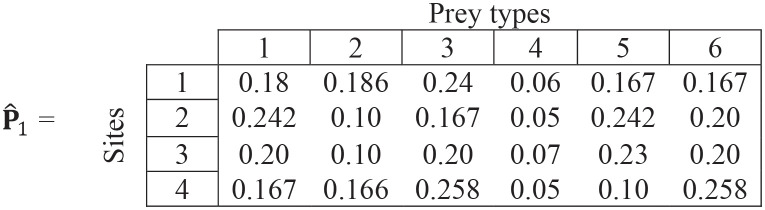
(17)

**Step (v)**. We estimate new overall values of overall preference by the mean of the values at each site. Thus overall preferences for all prey types at iteration #2 are:

$${\hat{\text{p}}}_{2}=\left[0.197\;0.138\;0.216\;0.057\;0.186\;0.206\right]$$ (18)

For example, the overall preference for prey type 1 (0.197) is the mean of the values in column 1 in matrix (17).

**Repeating steps (ii)–(iv)**

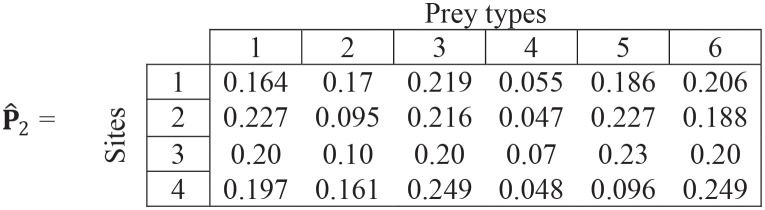
(19)

**Step (v)**.


$${\hat{\text{p}}}_{3}=\left[0.197\;0.132\;0.221\;0.055\;0.184\;0.211\right]$$ (20)

**Repeating steps (ii)–(iv)**

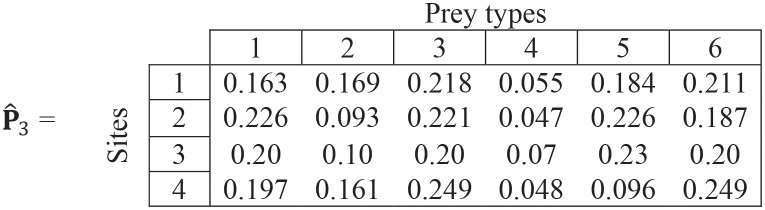
(21)

**Step (v)**.


$${\hat{\text{p}}}_{4}=\left[0.196\;0.131\;0.222\;0.055\;0.184\;0.212\right]$$ (22)

The overall preferences estimates show little changes between the 3^rd^ and 4^th^ iteration (Eqs (20) vs (22). Thus, the final estimate for overall preferences for each prey type is given by Eq (22).

## Simulation tests

### Methods

We tested the accuracy and precision of IPA using simulations. We varied the number of prey types, number of sites and the proportions of prey types missing per site. For each simulation, overall preferences (p_i_) were generated using a uniform distribution. Then mean prey density for each site was generated using a normal distribution with a mean of 100 and a coefficient of variation of 0.5, truncated to a minimum value of 5. Then the density of each prey type within each site was generated using a normal distribution with a mean of the site prey density and a coefficient of variation of 0.5. “Preference” was treated as the probability of choosing a prey item each time it was encountered. Thus the number of prey consumed for each prey type within each site was generated using a binomial distribution with a mean of p_i_ and an n of prey density. Finally, sites with incomplete collections of prey were simulated by using a binomial random number generator to randomly drop prey types from each site.

We ran simulations for all combinations of # of sites = 5, 10, 20, # of prey types = 4, 6, 10, 20, 50, and the proportion of missing prey types at each site = 0, 0.1, 0.2, 0.3, 0.4, 0.5, 0.6, .7, 0.8. We ran 100 replications for each combination of parameters. For each replication, we generated estimates of prey preference.

We then used bootstrapping to estimate confidence intervals with 100 bootstrapping samples. Confidence intervals were estimated using the bias-adjusted Studentized bootstrap [40].

We compared the IPA estimates to simply averaging preferences over all study sites (we will call this “Averaged Preference”). We calculated those indices for prey types at each site, and then calculated the mean for each prey type over all sites (while ignoring the missing prey types at each site).

## Results

**Fig 2:**
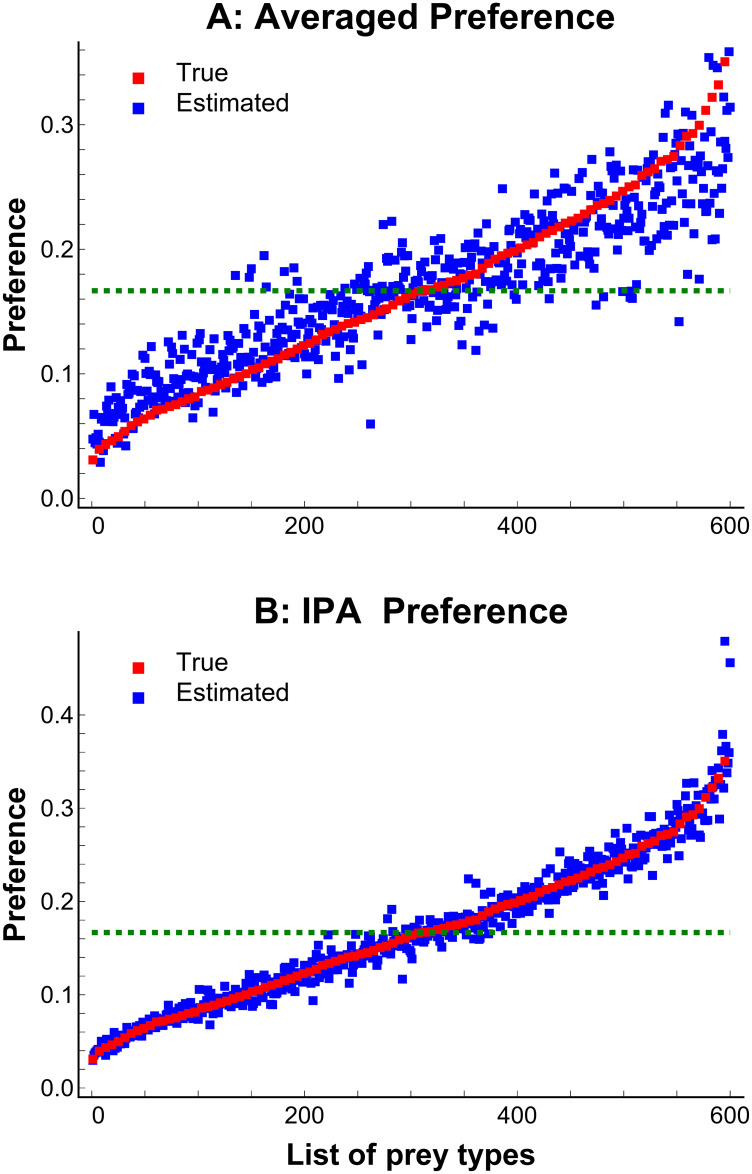
IPA is more accurate and precise than averaging. Simulation results comparing IPA to averaging. The list of prey types (x-axis) comprises 6 prey types x 100 replicates = 600 prey types, sorted by preference. (A) The averaged preference is simply averaging preferences over all study sites. (B) IPA is the method presented in this paper.

IPA gave more accurate and precise estimates of overall prey preference compared to the averaged preference. For example, Fig 2 shows the results of a simulation with 100 sites, 6 prey types, 50% of prey types present (varying from 10–90%) at each site, and 100 replicates. The averaged preference overestimates preference when preference is low and underestimates when preference is high. The lower precision and accuracy of the averaged estimates show the difficulty of estimating overall preference without considering missing prey types at each site.

**Fig 3:**
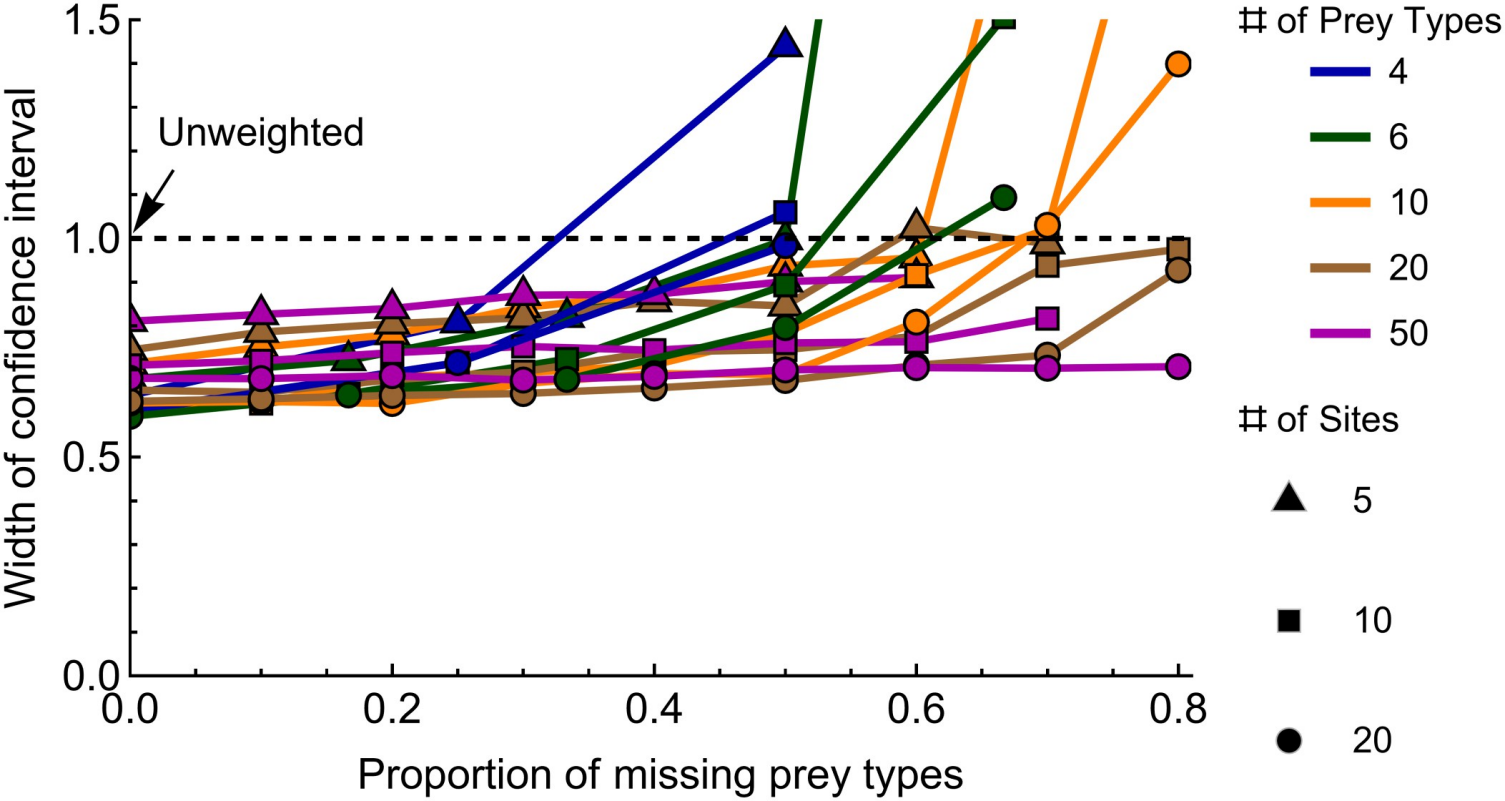
Weighting can increase precision of IPA estimates. Precision of weighted bootstrapped estimates of IPA are compared to unweighted estimates for simulated data. Smaller values mean a greater precision. Confidence intervals were estimated by bootstrapping 100 replications of each combination of parameters. Confidence interval width for the weighted estimate is divided by that for the unweighted—thus, values < 1 mean that precision of weighted is greater than the unweighted.

The IPA estimates are more precise when they are weighted by both prey densities and number of feeding samples for most combinations of parameters (Fig 3). Confidence intervals for weighted estimates are about 75% narrower than those for unweighted estimates. Weighting is more effective with a large number of sites. The proportion of missing prey types, the number of prey types and number of sites interact to affect precision. Precision is greater with fewer missing prey types, more prey types and more sites.

**Fig 4:**
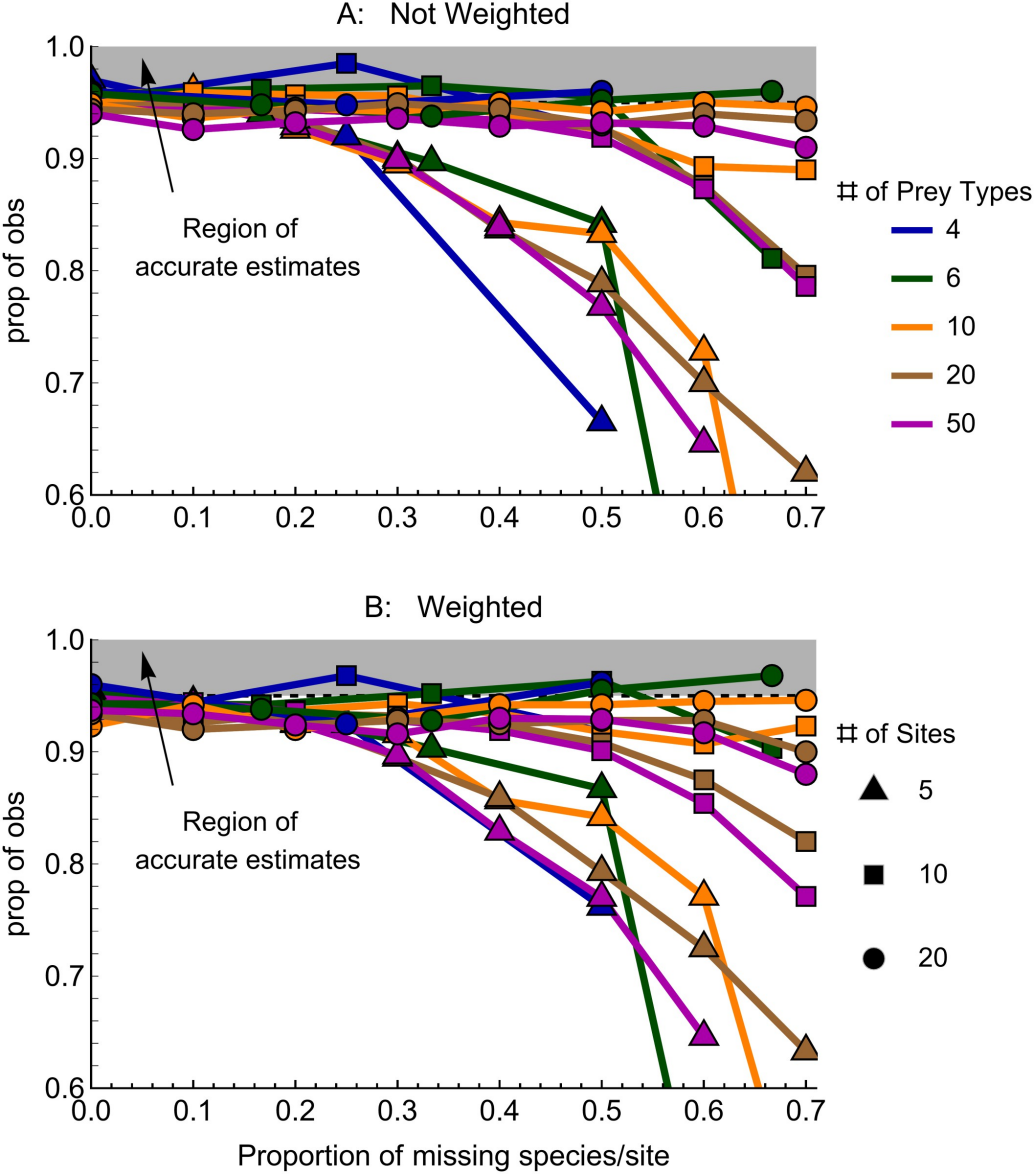
IPA gives accurate estimates when there are fewer prey types, smaller proportions of missing types per site, and more sites. Accuracy of weighted and unweighted bootstrapped estimates of IPA are estimated for simulated data. Confidence intervals were estimated by bootstrapping 100 replications of each combination of parameters. The Y axis is the proportion of results that are within 95% confidence intervals—thus, higher values are better. The gray region shows the proportion of results inside each 95% confidence interval—i.e. these are accurate estimates. There was no difference in accuracy between weighted and unweighted estimates.

Accuracy of bootstrapped estimates of confidence intervals varies with prey characteristics. The proportion of missing prey types, and the number of prey types and sites, all interact to affect accuracy. Generally, accuracy is greater with fewer missing prey types, more prey types, and more sites, for most combinations of parameters (Fig 4). There is little effect of weighting on accuracy. When there are more than 20 prey types, then bootstrapped confidence intervals are slightly too narrow—e.g. when there are less then 0.3 of prey types missing in each site, estimated 95% confidence intervals are actually like 90%.

## Case study

We illustrate the IPA method with lion feeding data [41] that includes 56 sites and 42 prey species. The high proportion of absent species at each site (0.7) should decrease accuracy and precision of the estimators, but the high number of sites and species should increase precision (Fig 3), and the high number of sites should increase accuracy (Fig 4). We estimated the overall preference of each prey type using both weighted and non-weighted estimators. To display the results, we ranked them according to prey weight and then fitted the general bell-shaped function:

$$pref=k_{3}{\text{e}}^{-\frac{{\left(wt-k_{1}\right)}^{2}}{k_{2}{}^{2}}}$$ (23)

where *k*_*1*_, *k*_*2*_, *k*_*3*_ = fitted parameters

*pref* = overall prey preference estimated by IPA

*wt* = log(prey weight).

Since the preferences are relative to each other, we scaled them so that the maximum function value would be 1—i.e. the preferences are all scaled relative to the maximum. We did not estimate the weighted IPA estimates because prey density and consumption surveys were not carried out in the same manner among sites.

Analysing overall preference by prey weights in this way yields several biological insights. First, we can describe feeding preferences by just using two parameters, representing the mean (k_1_) and standard deviation of the feeding curve (k_2_) (NB: not k_3_, since this is the same for all values of preference). k_1_ is a measure of average size of prey eaten, and k_2_ is a measure of feeding specialization (the width of the curve). If other predators fit similar curves, we can use those parameters to compare different predators and to estimate competition among predators living in the same system.

Second, we can estimate prey consumption by predators in systems with many prey types. Typically, potential prey consumption has been measured by classifying potential prey as either preferred vs non-preferred [42]–this assumes that the only preference choices are completely for or against. We can use this fitted curve to scale densities of each prey type according to preference, in order to get realistic estimates of prey consumption.

**Fig 5:**
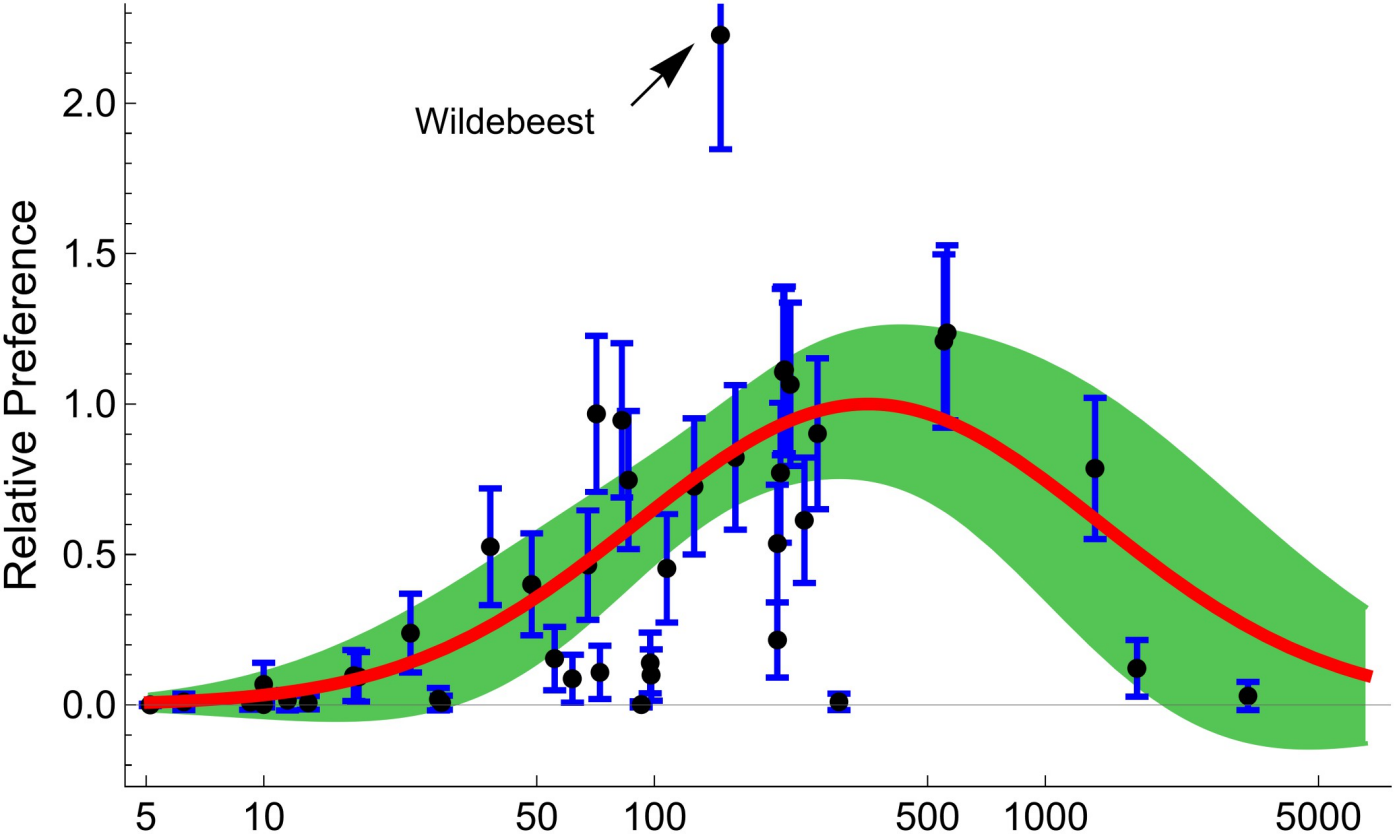
Lion prey preferences show a sigmoidal relationship based on prey weight. Prey preference is estimated for lion prey items from a series of study sites. Each dot represents one prey type and bars represent 95% confidence intervals. The solid red line represents a best-fit curve for a symmetrical curve of estimated relative overall preference vs log(prey weight) and the shaded areas represent 95% mean prediction bands.

Finally, we can identify prey types that merit more research. If overall preference for some prey type significantly deviates from the preference vs size curve, this means that the predator selects for that prey using other properties than just prey size. For example, lions prefer wildebeest (*Connochaetes* sp) more than any other prey species (Fig 5), and the confidence intervals for preference are outside of the preference vs size curve, showing that wildebeest are preyed upon more often than expected simply due to their size. This is supported by results showing that that wildebeest selection is also affected by rainfall [43].

## Discussion

### Sampling issues

There is some flexibility in how input data are gathered. The data input to IPA are prey abundances and consumption. But, because the method uses relative preferences, these data do not have to be estimated in the same way among sites—just the same within each site. Thus, one could estimate relative consumption based on scat analysis at one site and stomach contents at another site.

However, using the weighting when estimating sampling variation does somewhat limit application. IPA can use two kinds of weighting: based on prey densities and the total number of feeding samples in a site. Both types of sampling have to be carried out in the same way for all sites.

IPA overall preferences may need to be scaled appropriately, depending on how they are used. Overall preferences are relative to each other and sum to one. But if one wants to predict preferences in a new site with a more limited set of prey types, then those preferences no longer sum to one. Thus overall preferences need to be scaled according to the prey types available at that site, as follows:

If A = the set of prey items that are available in the new site, then the predicted preferences for prey type j in that study site are:

$$\frac{{\hat{\text{p}}}_{\text{j}}}{\sum_{\text{j}\in \text{A}}{\hat{\text{p}}}_{\text{j}}}$$ (24)

For example, suppose that there are 10 species, and overall preferences are estimated to be:

[0.15,0.05,0.2,0.05,0.24,0.2,0.03,0.03,0.01,0.04], (25)

and the new site has prey species {1,3,4,7,8} present. Then we would scale preferences for those species by 0.15+0.2+0.05+0.03+0.03, and get predicted preferences in the new site as:

[0.326,_,0.435,0.109,_,_,0.065,0.065,_,_] (26)

The preferences need to be scaled differently for consumption estimation. In order to estimate overall prey consumption, consumption = preference × density, and thus the maximum possible preference would be 1. If you assume that the most preferred prey in that site is effectively always chosen, then you would scale all the preferences by dividing by the maximum value. Using the above example, we would divide the values for those species from (25), by the maximum, 0.2, and get predicted preferences in the new site as:

[0.749,_,1,0.251,_,_,0.149,0.149,_,_] (27)

To estimate prey consumption, one would multiply the values in (27) by prey densities.

### Preference indices

IPA uses Manly’s α for the index of prey preference. Many other studies have used the Jacobs’ index (e.g. [18, 41, 44]). See S1 Appendix for how IPA can incorporate the Jacobs’ index. These indices differ in many ways [37]:

1. Manly’s ∝ ranges from (0,1) and Jacobs’ index from (-1, 1).
2. “No preference" for Manly’s ∝ is 1/(# of prey types), while for Jacobs’ index it is 0. Consequently, for Manly’s ∝ the range of prey preferences for avoidance is smaller than for selection whereas for Jacobs’ the range of prey preferences for avoidance vs selection is the same.
3. Jacob’s index is affected by relative prey densities when there are more then two prey types [24, 45]–thus, one can only compare preferences in different situations if prey densities are the same. Manly’s ∝ is independent of prey densities.
4. The total of Manly’s ∝ for all prey types is 1 but there is no constant total for Jacobs’ index.
5. All preference indices are relative. If preferences are estimated for a large suite of prey items, then one cannot just apply those values directly in a different situation with fewer prey. However Manly’s ∝ can be re-normalized to take into account the dropping of prey items

IPA uses Manly’s α because IPA hinges on scaling the preference measures to those you would expect if all prey types were present in each site (Step 1). The scaling requires that prey preferences range from 0 to 1, and sum to 1 in each site—and Manly’s α is the only published index that does this.

However, Manly’s α has a weakness in that it is sensitive to variations in densities of rare prey. IPA minimizes this problem in two ways: first, mean preference (Step 3) is weighted by prey density, and second, when estimating sampling error during bootstrapping, the preferences are transformed by arcsine(2 x − 1).

On the other hand, Manly’s ∝ is useful when predicting total biomass of prey consumed—one simply multiplies Manly’s ∝ by prey density and body mass. In addition, Manly’s ∝ is biologically relevant in that it represents the relative probability of eating a prey once it is encountered [23].

### Availability biases

When organism densities are estimated, several availability biases can occur, and these can potentially affect preference estimates. False absences occur when individuals are present but undetected, and false presences occur when individuals are not present but are wrongfully identified to be present [46]. If such biases underestimate prey availability, then preferences are overestimated. However, IPA is only affected indirectly by such biases, since it uses estimates of prey availability, but does not specify how those estimates should be obtained. Thus, prey availability estimates can be made using one of the many density estimation methods that minimize such biases [37].

### Ecological differences among sites

If important ecological differences exist among sites, then this might affect the accuracy of overall estimates of prey preference, and thus of conservation interventions. There are two ways of dealing with this. First, find out if preferences do differ among sites, by comparing standardized preferences within each site (i.e. step (iv)) to overall preferences. Currently this would be done visually, and thus work is needed to develop statistical tests for this. Sites that are different would then be treated separately. Second, instead of analyzing preference based on prey species, one could use prey weights. Many predators select prey mostly based on size, not prey species [27]. The lion example discussed above illustrates this.

### Applications

IPA can be generalized. In the description of IPA, we use the term “prey types”; prey can be categorized in many ways. For example, Lungdoh *et al*. [44] classified prey using broader taxonomic units than species. Or one might use some features of the prey, such as size or microhabitat use. These features could be revealed using IPA. Then, one could classify prey according to that feature and then use IPA to generate final estimates of overall preference. We can also generalize the resource; IPA can be used for any type of resource, such as habitats or nest sites.

In studies of predator-prey relationships, IPA can also be used to estimate overall prey consumption. Many of the key studies of predator-prey relationships have focused on systems with a limited number of main prey types (for example, the snowshoe hare (*Lepus americanus*)–lynx (*Lynx canadensis*) system [47] and the wolf (*Canis lupus*) -moose (*Alces alces*) system [48]), because it is difficult to estimate overall prey consumption when there are many prey types. When researchers have studied systems with many prey types, they have had to make simplifying assumptions. For example, Lindsey *et al*. [49] compared cheetah (*Acinonyx jubatus*) prey requirements in 12 South African game reserves in order to manage re-introductions of cheetah. To combine data from many sites, the researchers had to focus on only the two most important prey types. Using IPA, we can estimate relative prey preferences for all prey types and thus combine them into one overall measure of prey consumption. Also, if we include prey weight, we can estimate overall biomass of prey consumed. This flexibility will make it easier to build predator-prey models with many varied prey types.

One can use IPA to test hypotheses about factors that affect prey preference. The type of test depends on whether the factors are inherent properties of the prey types or ones that can vary among sites. For example, some properties that are inherent are handling time, risk of injury during capture, taste and size of the prey [41]. Some properties that vary among sites are types of habitats, and prey abundance and diversity [41]. Using IPA, we can test for the inherent properties by comparing overall estimated preferences among different prey types. A strength of using IPA to do this is that estimating preferences over many sites ensures that effects of the varying properties should average out. Thus, the more, and different, sites, the better.

We can test for the varying properties by analyzing the differences between estimated and observed preferences for each prey type and site. IPA preferences are estimated under the assumption of constant preferences among sites, and thus can be viewed as a null hypothesis. Models can be tested predicting the differences between estimated and observed preferences as a function of prey density, prey diversity, or various habitat features. This allows us to test hypotheses that had previously been limited to a narrow range of study sites. For example, Prugh [7] showed that coyotes (*Canis latrans*) change prey preference in response to changes in snowshoe hare (*Lepus americanus*) density. Such comparisons were possible because Prugh worked within one study site over several years, with a relatively constant number of prey types. Such an analysis can now be carried out using IPA over a wide range of study sites that differ in prey types. As of July 2019, there are at least 109 studies of coyote feeding habits that could be used in such a comparison.

Application of IPA allows us to: test hypotheses about what factors affect prey selection, predict preferences in new sites, and estimate overall prey consumption in new sites. The method’s robust flexibility could lead to a general theory of feeding preference that will allow us to understand and predict food choice.

## Supporting information

S1 Appendix Using IPA to estimate the Jacob’s preference index. (DOCX)

## References

[1] Pyke GH, Pulliam MR, Charnov EL. Optimal foraging: a selective review of theory and tests. Q Rev Biol. 1977;52: 137–154.

[2] Perry G, Pianka ER. Animal foraging: past, present and future. Trends Ecol Evol. 1997;12: 360–364. doi: 10.1016/s0169-5347(97)01097-5 21238109

[3] Brown JS, Laundré JW, Gurung M. The Ecology of Fear: Optimal Foraging, Game Theory, and Trophic Interactions. J Mammal. 1999;80: 385–399. doi: 10.2307/1383287

[4] Endler JA. Frequency-dependent predation, crypsis and aposematic coloration. Philos Trans R Soc Lond Ser B-Biol Sci. 1988;319: 505–523. doi: 10.1098/rstb.1988.0062 2905489

[5] Ishii Y, Shimada M. Learning predator promotes coexistence of prey species in host–parasitoid systems. Proc Natl Acad Sci. 2012;109: 5116–5120. doi: 10.1073/pnas.1115133109 22411808 PMC3324012

[6] Oaten A, Murdoch WW. Switching, functional response, and stability in predator-prey systems. Am Nat. 1975;109: 299–318.

[7] Prugh LR. Coyote prey selection and community stability during a decline in food supply. Oikos. 2005;110: 253–264. doi: 10.1111/j.0030-1299.2005.13478.x

[8] Kennedy M, Gray R. Can Ecological Theory Predict the Distribution of Foraging Animals? A Critical Analysis of Experiments on the Ideal Free Distribution. Oikos. 1993;68: 158–166.

[9] Křivan V, Cressman R, Schneider C. The ideal free distribution: A review and synthesis of the game-theoretic perspective. Theor Popul Biol. 2008;73: 403–425. doi: 10.1016/j.tpb.2007.12.009 18282592

[10] Holling CS. The Functional Response of Invertebrate Predators to Prey Density1. Mem Entomol Soc Can. 1966;98: 5–86. doi: 10.4039/entm9848fv

[11] Kalinkat G, Schneider FD, Digel C, Guill C, Rall BC, Brose U. Body masses, functional responses and predator–prey stability. Ecol Lett. 2013;16: 1126–1134. doi: 10.1111/ele.12147 23819684

[12] Zimmermann B, Sand H, Wabakken P, Liberg O, Andreassen HP. Predator-dependent functional response in wolves: from food limitation to surplus killing. J Anim Ecol. 2014; 102–112. doi: 10.1111/1365-2656.12280 25109601

[13] Mattioli L, Capitani C, Gazzola A, Scandura M, Apollonio M. Prey selection and dietary response by wolves in a high-density multi-species ungulate community. Eur J Wildl Res. 2011;57: 909–922. doi: 10.1007/s10344-011-0503-4

[14] Sebastiano S, Antonio R, Fabrizio O, Dario O, Roberta M. Different season, different strategies: Feeding ecology of two syntopic forest-dwelling salamanders. Acta Oecologica. 2012;43: 42–50. doi: 10.1016/j.actao.2012.05.001

[15] Davidson Z, Valeix M, Kesteren FV, Loveridge AJ, Hunt JE, Murindagomo F, et al. Seasonal Diet and Prey Preference of the African Lion in a Waterhole-Driven Semi-Arid Savanna. PLOS ONE. 2013;8: e55182. doi: 10.1371/journal.pone.0055182 23405121 PMC3566210

[16] Estlander S, Nurminen L, Olin M, Vinni M, Immonen S, Rask M, et al. Diet shifts and food selection of perch Perca fluviatilis and roach Rutilus rutilus in humic lakes of varying water colour. J Fish Biol. 2010;77: 241–256. doi: 10.1111/j.1095-8649.2010.02682.x 20646150

[17] Cavalli M, Baladrón AV, Isacch JP, Martínez G, Bó MS. Prey selection and food habits of breeding Burrowing Owls (Athene cunicularia) in natural and modified habitats of Argentine pampas. Emu—Austral Ornithol. 2014;114: 184–188. doi: 10.1071/MU13040

[18] Hayward MW, Jędrzejewski W, Jêdrzejewska B. Prey preferences of the tiger P anthera tigris. J Zool. 2012;286: 221–231. doi: 10.1111/j.1469-7998.2011.00871.x

[19] Hayward MW, Kamler JF, Montgomery RA, Newlove A, Rostro-García S, Sales LP, et al. Prey Preferences of the Jaguar Panthera onca Reflect the Post-Pleistocene Demise of Large Prey. Front Ecol Evol. 2016;3.

[20] Hayward MW, Porter L, Lanszki J, Kamler JF, Beck JM, Kerley GIH, et al. Factors affecting the prey preferences of jackals (Canidae). Mamm Biol. 2017;85: 70–82. doi: 10.1016/j.mambio.2017.02.005

[21] Ivlev VS. Experimental ecology of the feeding of fishes. New Haven: Yale University Press; 1961.

[22] Manly BFJ, Miller P, Cook LM. Analysis of a selective predation experiment. Am Nat. 1972;106: 719–736.

[23] Chesson J. Measuring preference in selective predation. Ecology. 1978;59: 211–215.

[24] Lechowicz MJ. The sampling characteristics of electivity indices. Oecologia Berl. 1982;52: 22–30. doi: 10.1007/BF00349007 28310104

[25] Alldredge JR, Ratti JT. Comparison of some statistical techniques for analysis of resource selection. J Wildl Manag. 1986;50: 157–165.

[26] Hayward MW. Prey preferences of the spotted hyaena (Crocuta crocuta) and degree of dietary overlap with the lion (Panthera leo). J Zool. 2006;270: 606–614. doi: 10.1111/j.1469-7998.2006.00183.x

[27] Hayward MW, Kerley GIH. Prey preferences and dietary overlap amongst Africa’s large predators. South Afr J Wildl Res. 2008;38: 93–108. doi: 10.3957/0379-4369-38.2.93

[28] Johnson DH. The comparison of usage and availability measurements for evaluating resource preference. Ecology. 1980;61: 65–71.

[29] Wolfram Research. Mathematica. Champaign, Illinois: Wolfram Research, Inc.; 2021.

[30] Nams VO, Garnett S. Code for IPA algorithm, V1.1. V1.1. 2020.

[31] Spaan D, Ramos-Fernández G, Schaffner CM, Smith-Aguilar SE, Pinacho-Guendulain B, Aureli F. Standardizing methods to estimate population density: an example based on habituated and unhabituated spider monkeys. Biodivers Conserv. 2019;28: 847–862. doi: 10.1007/s10531-018-01696-2

[32] Elliot NB, Gopalaswamy AM. Toward accurate and precise estimates of lion density. Conserv Biol. 2017;31: 934–943. doi: 10.1111/cobi.12878 27958641

[33] Nams V, Gillis E. Changes in tracking tubes use by small mammals over time. J Mammal. 2003;84: 1374–1380.

[34] Moran Z, Orth DJ, Schmitt JD, Hallerman EM, Aguilar R. Effectiveness of DNA barcoding for identifying piscine prey items in stomach contents of piscivorous catfishes. Environ Biol Fishes. 2016;99: 161–167.

[35] du Preez B, Purdon J, Trethowan P, Macdonald DW, Loveridge AJ. Dietary niche differentiation facilitates coexistence of two large carnivores. J Zool. 2017;302: 149–156. doi: 10.1111/jzo.12443

[36] Floyd TJ, Mech LD, Jordan PA. Relating wolf scat content to prey consumed. J Wildl Manag. 1976;42: 528–532.

[37] Krebs CJ. Ecological Methodology. Second. New York: Harper & Row; 1999.

[38] Efron B, Tibshirani RJ. An introduction to the bootstrap. CRC press; 1994.

[39] Lohr SL. Sampling: Design and Analysis. 3rd edition. Boca Raton: Chapman and Hall/CRC; 2021.

[40] Manly BFJ. Randomization, Bootstrap and Monte Carlo Methods in Biology. 3rd ed. Boca Raton, FL: Chapman and Hall/CRC; 2006.

[41] Hayward MW, Kerley GIH. Prey preferences of the lion (Panthera leo). J Zool. 2005;267: 309–322. doi: 10.1017/S0952836905007508

[42] Hayward MW, O’Brien J, Kerley GIH. Carrying capacity of large African predators: Predictions and tests. Biol Conserv. 2007;139: 219–229. doi: 10.1016/j.biocon.2007.06.018

[43] Owen-Smith N. Changing vulnerability to predation related to season and sex in an African ungulate assemblage. Oikos. 2008;117: 602–610. doi: 10.1111/j.0030-1299.2008.16309.x

[44] Lyngdoh S, Shrotriya S, Goyal SP, Clements H, Hayward MW, Habib B. Prey Preferences of the Snow Leopard (Panthera uncia): Regional Diet Specificity Holds Global Significance for Conservation. PLOS ONE. 2014;9: e88349. doi: 10.1371/journal.pone.0088349 24533080 PMC3922817

[45] Jacobs J. Quantitative measurement of food selection. Oecologia. 1974;14: 413–417. doi: 10.1007/BF00384581 28308662

[46] Southgate R, Dziminski MA, Paltridge R, Schubert A, Gaikhorst G. Verifying bilby presence and the systematic sampling of wild populations using sign-based protocols–with notes on aerial and ground survey techniques and asserting absence. Aust Mammal. 2018;41: 27–38.

[47] Krebs CJ, Boutin S, Boonstra R, Sinclair ARE, Smith JNM, Dale MRT, et al. Impact of food and predation on the snowshoe hare cycle. Science. 1995;269: 1112–1115. doi: 10.1126/science.269.5227.1112 17755536

[48] Peterson RO. Wolf-moose interaction on Isle Royale: The end of natural regulation? Ecol Appl. 1999;9: 10–16.

[49] Lindsey P, Tambling C j., Brummer R, Davies-Mostert H, Hayward M, Marnewick K, et al. Minimum prey and area requirements of the Vulnerable cheetah Acinonyx jubatus: implications for reintroduction and management of the species in South Africa. Oryx. 2011;45: 587–599. doi: 10.1017/S003060531000150X

