# Supplementary material for: A method to predict overall food preferences
Source: PLoS One. 2022 Jun 3;17(6):e0268520. doi: 10.1371/journal.pone.0268520 (PMC9165852; doi:10.1371/journal.pone.0268520)
Supplement: S1 Appendix — (DOCX) [file pone.0268520.s001.docx]

# S1 Appendix. Preference indices

The first preference ever index proposed was Ivlev's electivity index [3]:

${\text{E}_{\text{j}}}_{\text{ }}= \frac{e_{\text{j}}}{d_{\text{j}}}$,

(1)

where d_j_ = relative density of prey item j, and

e_j_ = relative amount of prey item j.

While this index seems intuitively obvious, one flaw is that it is affected by relative prey densities. Two indices have been proposed to avoid this flaw. Manly's ∝ [1] is independent of relative densities for any number of species[2], and Jacobs' index for only 2 prey species [4,5]. Manly's ∝ is simply the normalized Ivlev's E:

| $\propto_{\text{j }}=$ | $\frac{e_{\text{j}}}{d_{\text{j}}}$ |
| --- | --- |
|  | $\sum_{j} \frac{e_{\text{j}}}{d_{\text{j}}}$ |

(2)

And Jacobs' index is:

$${\text{D}_{\text{j}}}_{\text{ }}= \frac{e_{\text{j}}-d_{\text{j}}}{e_{\text{j}}+d_{\text{j}}-2e_{\text{j}}d_{\text{j}}}$$

(3)

Since both indices are useful for different situations, it would be useful for the IPA method to estimate them. We do this as follows. Since the Jacobs index is dependent on prey density (if there are more than 2 prey types), there is no general transformation between these two indices without knowing the actual prey densities. However, in the special case where all prey densities are equal, the Manly’s ∝ is:

∝_j_ = *e_j_*

(4)

and Jacobs' index is:

$${\text{D}_{\text{j}}}_{\text{ }}= \frac{e_{\text{j}}- \frac{1}{n}}{e_{\text{j}}+\frac{1}{n}-2e_{\text{j}}\frac{1}{n}}$$

(5)

where n = number of prey items.

If we combine equations (4) and (5) and rearrange, we can estimate Jacobs’ (we will call this transformed estimate, JacobsT) from Manly’s by:

$${\text{D}_{\text{j}}}_{\text{ }}= \frac{n \propto_{j}-1}{1+(n-2)\propto_{j}}$$

(6)

Note that the Jacobs’ index is dependent on prey densities when there are more than two prey types. But since JacobsT is estimated from the Manly’s index, which is not dependent on prey densities, thus JacobsT is not dependent on prey densities.

We incorporate Jacobs' index into IPA by calculating it at the end of the iterations, before bootstrapping.

# References

1. Manly BFJ, Miller P, Cook LM. Analysis of a selective predation experiment. Am Nat. 1972;106: 719–736.

2. Chesson J. Measuring preference in selective predation. Ecology. 1978;59: 211–215.

3. Ivlev VS. Experimental ecology of the feeding of fishes. New Haven: Yale University Press; 1961.

4. Jacobs J. Quantitative measurement of food selection. Oecologia. 1974;14: 413–417. doi:10.1007/BF00384581

5. Lechowicz MJ. The sampling characteristics of electivity indices. Oecologia Berl. 1982;52: 22–30.
